# Inflammation, Biomarkers and Immuno-Oncology Pathways in Pancreatic Cancer

**DOI:** 10.3390/jpm9020020

**Published:** 2019-04-26

**Authors:** Belinda Lee, Peter Gibbs

**Affiliations:** 1 Division of Systems Biology & Personalized Medicine, Walter & Eliza Hall Institute (WEHI), Parkville, Victoria 3052, Australia; peter.gibbs@mh.org.au; 2 Department of Medical Biology, University of Melbourne, Parkville, Victoria 3010, Australia; 3 Department of Medical Oncology, Peter MacCallum Cancer Centre, Parkville, Victoria 3000, Australia; 4 Department of Medical Oncology, The Northern Hospital, Epping, Victoria 3076, Australia

**Keywords:** pancreatic cancer, pancreatic ductal adenocarcinoma (PDAC), immune microenvironment, immune biomarkers, personalized cancer care, inflammation, PD1, CTLA-4

## Abstract

It is estimated that pancreatic cancer will be the second leading cause of cancer-related deaths globally by 2030, highlighting the ongoing lack of effective treatment options for this devastating condition. There is a lack of reliable prognostic or predictive markers in pancreatic cancer to guide management decisions, whether for systemic chemotherapy, molecularly targeted therapies, or immunotherapies. To date, the results for targeted agents and immunotherapies in unselected populations of chemo-refractory pancreatic cancer have not met expectations. The reasons for this lack of efficacy of immunotherapy in pancreatic cancer are not completely understood. The challenges in pancreatic cancer include the physical barrier created by the dense desmoplastic stroma surrounding the tumor, chemokine-mediated exclusion of T cells, relatively poorer antigenicity compared to other solid tumors, paucity of infiltrating T cells within the tumor, ultimately leading to an immunosuppressive microenvironment. A better understanding of the role of inflammation in pancreatic cancer, its tumor microenvironment and individualized patient-related features, be they molecular, clinical or histopathological, would enable a more effective tailored approach to the management of pancreatic cancer. In this review, the role of inflammation, the immune tumor microenvironment and potential immune biomarkers in pancreatic cancer are explored.

## 1. Introduction

Pancreatic cancer currently has the lowest survival rate of all cancers. Only 8 out of every 100 patients diagnosed with pancreatic cancer survives beyond 5 years [1]. Recent published data looking at outcomes from 2005 to 2015 demonstrate no improvements in survival outcomes in the last 10 years [2]. Pancreatic cancer is currently the fifth most common cause of cancer-related death in the United States, but it is projected by 2030 to become the second leading cause of cancer-related death globally [3]. These statistics highlight the need for not only better therapies but also more durable treatment responses. 

The most common form of pancreatic cancer is pancreatic ductal adenocarcinoma (PDAC). Pancreatic ductal adenocarcinomas are stromal-rich tumors that develop within the exocrine compartment of the pancreatic gland. Pancreatic ductal adenocarcinoma accounts for more than 85% of all pancreatic malignancies [4]. Early stage or resectable disease occurs in just 20% of cases, 35–40% have locally advanced unresectable disease and 45–55% have metastatic disease at presentation [3]. Factors contributing to more advanced stage at diagnosis include the non-specific nature of the presenting symptoms, the lack of good biomarkers to identify patients at risk of the disease, and the retroperitoneal location of the pancreas, which limits routine tissue sampling and radiographic screening.

A unifying feature of all pancreatic tumors is the presence of immune and fibroblast cells, suggesting a critical role for inflammation in tumor progression. The impermeable desmoplastic stroma found around the pancreatic tumor is hypothesised to prevent immune cell infiltration and hinder effective delivery of chemotherapy [5,6]. It has also been suggested that abnormal stromal activation contributes to tumorigenesis and further immunosuppression, hypoxia and an anti-angiogenic tumor microenvironment [5]. One proposed model suggests that PDAC evolution occurs from PanIN 1 through to PanIN3 and then to invasive PDAC with progressive accumulation of desmoplastic stroma and modulation of the character of leucocyte infiltration altering through this process [7]. Tumor associated macrophages (TAM) are reported to increase early in PDAC tumorigenesis, with reduction or varying levels of neutrophils and T cells present in PanIN and PDAC [8]. 

Genomic alterations are also implicated in the development of PDAC and may play a role in the immunogenicity of individual tumors. Recurrent genetic damage in 10 core molecular pathways involving over 40 genes has been identified in PDAC [9,10]. The most commonly occurring mutations in PDAC include KRAS in 75–90%, TP53 in 50–80%, CDKN2A in 49–98%, SMAD4 in 19–50%, and BRCA1 and BRCA2 in 4–14% of PDAC tumors [8,11]. Pancreatic ductal adenocarcinoma typically has a relatively low mutation burden at 3–4/ mutations per base (MB) which may partly account for the poorer antigenicity compared to other solid tumors. However, the highly immunosuppressive tumor microenvironment (TME) plays a significant role in hindering T-cell activation, which in turn contributes to the general lack of response to immunotherapy [12,13]. Six immune subtypes have been identified across a range of solid tumors—immunologically quiet, lymphocyte deplete, inflammatory, wound healing, IFNγ dominant, and TGFβ dominant [14]. These subtypes appear to be governed by variations in immune cells—namely, macrophage and lymphocyte signatures, Th1:Th2 cell ratio, tumor heterogeneity and genomic-based differences of aneuploidy, neo-antigen load, cell proliferation, and expression of immunomodulatory genes [14,15]. 

More specifically for PDAC tumors, genomic immune analysis has identified a predominantly inflammatory immune signature that is defined by raised levels of TH17 and Th1 genes, as well as a low-to-moderate tumor cell proliferation [14]. Not surprisingly, a low leucocyte proportion of tumor stromal fraction of <10% was reported. In an analysis by Knudsen and colleagues, a cohort of 109 PDAC tumors were sequenced and analysed to determine the composite relationship of neoantigens and stromal features [16]. This study reported four distinct immune clusters in PDAC. Cluster 1 exhibited a low number of mutations, low stromal volume, an “immature” or higher cellular but collagen-poor stromal compartment, and a predominantly macrophage immune cell infiltrate. Cluster 2 demonstrated high levels of all immune cell subsets and a higher number of mutations. Cluster 3 reported a high mutational burden and an intermediate stromal compartment morphology, greater number of tumor infiltrating lymphocytes and peritumoral lymphocytes, but lower levels of CD68 and CD163 macrophages. Cluster 4 exhibited reduced levels of all immune and stromal markers with a “mature” or dense collagenous stromal compartment, high stromal volume, low neo-antigen levels and the presence of a KRAS mutation but lack of other canonical genetic events such as CDKN2A, MYC and TP53, which were underrepresented in cluster 4. In both uni- and multi-variate analysis, considering tumor grade and lymph node status, cluster 4 was associated with significantly better overall survival. 

Other contributing factors that influence tumor-immune interactions in this complex intracellular and extracellular messaging network include differences in copy number, epigenetic changes, microRNAs and transcription [14,15]. Pancreatic ductal adenocarcinoma tumors with higher copy number alterations (aneuploidy) have greater chromosomal instability and exhibit more mutations in DNA break repair genes. Aneuploidy is an independent factor for poor prognosis in PDAC [17]. The presence of aneuploidy has been shown to reduce expression of cytokines -IFNg, IL-1A, IL-1B, and IL-2, which are responsible for tumor destruction [18]. Compared to the mutation number, increased copy number alterations demonstrated a stronger association with the inflammatory cytotoxic immune signature and markers of immune evasion [18].

Recent advancements in genomic technologies have led to the identification of aberrant activated epigenetic pathways and microRNA signatures in PDAC. These epigenetic pathways are responsible for driving growth signaling, silencing tumor suppression pathways and cell-cycle checkpoints. Epigenetic modifications, including DNA methylation and histone post-translation modifications, are present across a range of tumors, including PDAC [19,20]. Examples of epigenetic modifications that influence the tumor-immune interaction include loss of tri-methylation on histone 3, lysine 9 (H3K9) and H3K4me3. These epigenetic modifications are reported to mediate PDAC formation and immune evasion, and correlate with increased PDAC metastatic progression, and poorer prognosis, respectively. The H3K4me, H3K9me and H3K27me epigenetic pathways and their respective enzymatic targets (KDM1A, G9a and SUV39H1, and EZH2) have demonstrated in the preclinical setting the possibility of reprogramming or “resetting” the TME and increasing drug responsiveness [19,20,21]. These epigenetic marks are now being incorporated into early phase clinical trials (NCT02929498, NCT02395601 and NCT03009344). 

## 2. Current Treatment Paradigm

Pancreatectomy remains the single most effective treatment modality for the management of early stage disease, and the only potential for cure [22]. However, even with “curative” resection and adjuvant therapy, the 5-year survival rate of early stage resected PDAC remains limited to 20–25% [23]. For patients with advanced disease at presentation, the outlook is even more dismal with systemic therapy offering only a modest benefit [24]. The use of single agent gemcitabine chemotherapy achieves survival rates of only 18% at 1 year and 2% at 5 years [24]. The median survival with locally advanced disease ranges from 9 to 15 months, whereas for those with metastatic disease it is as low as 3–6 months [3,4]. 

In recent years, the use of two combination chemotherapy regimens has demonstrated superior efficacy over single agent gemcitabine. The use of FOLFIRINOX (a combination of oxaliplatin, irinotecan and 5 fluorouracil) in the advanced PDAC setting extended the median overall survival (OS) to 11 months, whereas the combination of albumin bound paclitaxel (Abraxane) with gemcitabine extended the median OS to 8 months [25,26]. Both these chemotherapy combinations are accepted as standard first-line palliative options in the advanced PDAC disease setting but only offer limited benefit. Regardless of these treatments and the choice of first-line chemotherapy, the overall 5-year survival rate for pancreatic cancer is less than 8% [4]. Sadly, disease progression for pancreatic cancer patients with locally advanced or metastatic disease is inevitable. 

There is a clear need to step outside the current treatment paradigm and investigate new diagnostic, monitoring and therapeutic opportunities to improve outcomes for this devastating disease, in both early and late-stage pancreatic cancer. The results to date in early-phase clinical trials of novel targeted agents in unselected chemo-refractory pancreatic cancer have fallen short of the anticipated efficacy, highlighting the lack of a reliable validated predictive or prognostic biomarker in pancreatic cancer that can inform and guide treatment decisions. A more selective approach, by identifying and utilizing biomarkers that characterize patients, could enable greater benefit from current and novel therapies. 

## 3. Immune Biomarkers in PDAC

Characterization of immune cells within the TME was first described by Virchow in 1863; since then, work to immune-phenotype the TME of solid tumors has progressed using immune cell-specific antibodies, multiplex immunohistochemistry and most recently spatial transcriptomics. There are limited published reports on the presence of tumor-infiltrating lymphocytes (TILs) assessed on H&E sections in PDAC. One study by Hart et al examined H&E sections from 63 PDAC patients and reported no difference in survival outcomes between patients with a high versus a low intra-tumoral lymphocyte score [27]. In comparison, the presence of intra-tumoral tertiary lymphoid organs/follicles is associated with longer disease-free survival (DFS) and OS [28]. 

A range of immune cell markers have been investigated in PDAC with good prognostic associations found for higher expression of CD3, CD4, CD8, and CD20 [29,30]. In comparison, higher expression of FOXP3, CD66b, CD68, CD163, and CD204 is associated with worse outcomes [29]. Myeloid cell infiltrates appear to dominate the immune reaction in PDAC [29,31]. Elevated levels of circulating myeloid-derived suppressor cells (MDSC) and regulatory T cells (Tregs) have been reported in PDAC patients, and are associated with worse prognosis and OS [31].

The mere presence of T cells within the PDAC tumor may have limited clinical correlation. The expansion of tumor-infiltrating CD3+ T cells isolated from resected PDAC specimens confirmed that a subset of T cells is present within the tumor and has tumor reactivity; however, some of these T cells have lost CD3zeta expression required for T cell receptor signaling and activation or lack the T-cell activation gene signature. These T cells are considered to be hypo-functioning [32]. Thus, characterizing the type of immune cells, density and location of immune cells in the PDAC is crucial to filling the gaps in our current knowledge and determining the therapeutic targeting and potential of novel immunotherapies [33].

Of interest are immune biomarkers that could potentially be used to guide treatment choices with novel immunotherapies, as they can be monitored easily, and cheaply, by blood-based screening. Inflammatory cytokines promote PDAC growth and progression both through direct stimulation of tumor cells as well as by modifying the TME [34,35]. Work by Piro et al. into the relationship between circulating Th2 cytokines and long-term survival in early-stage resectable PDAC has demonstrated that a simultaneous low expression of interleukin (IL)4 and high expression of TNFα is associated with better DFS following surgery (HR = 0.313, *p* < 0.0001) [35]. In contrast, elevated levels of TH2 cytokines—IL4, IL5, IL6, macrophage inflammatory protein (MIP)1α, granulocyte-macrophage colony stimulating factor (GM-CSF) and monocyte chemoattractant protein (MCP)1 and IL17, IFNɤ-induced protein (IP)10 and IL1b—were significantly associated with a shorter DFS [35]. Based on these findings, Piro et al. have suggested that IL4 is an independent prognostic factor for DFS in PDAC, and assessing this biomarker prior to surgery could potentially identify patients most at risk of early recurrence. 

Other cytokines, including elevated IL6, CRP and macrophage inhibitory cytokine (MIC)-1, have also been identified as negative prognostic biomarkers for OS in PDAC [36]. An attempt to apply a combined scoring for C-reactive protein (CRP) and the pancreatic Glasgow Prognostic Score (GPS) to predict outcomes in the phase 2 trial evaluating capecitabine with and without ruxolitinib (JAK/STAT inhibitor) showed initial promise but was not successful in the subsequent phase 3 trial [37]. A more in-depth understanding of the relationship between cytokines and therapeutic response is still required. Research combining circulating plasma cytokine levels with tumor RNA sequencing could help to shed more light on the complex immune-tumor relationship in PDAC. 

To date, the administration of immunotherapy in clinical trials in PDAC has not demonstrated any substantial benefit for patients with pancreatic cancer [38,39,40]. The reasons for this are not completely understood but are likely to be multifactorial. Proposed mechanisms leading to immunotherapy resistance in PDAC include a markedly immunosuppressive and “immune-privileged” tumor microenvironment [29]. 

Pancreatic stellate cells (PSC) have been identified as a primary source of excessive extracellular matrix [41], whereas activated PSCs with a high expression of galectin-1 appear to influence the development and maintenance of the immunosuppressive TME and pancreatic cancer immune evasion [42]. Activated PSCs are reported to form a barrier that prevents CD3+ T cells from infiltrating the pancreatic cancer tissue, they also stimulate T cell apoptosis, decrease IL2, and decrease IFNɤ Th1 secretion, while increasing immune suppressive IL4, IL5 Th2 cytokine secretion [42]. These findings suggest that galectin-1 expression on PSCs plays a crucial role in T-cell response and immunosuppression within the PDAC TME, and are consistent with clinical correlative studies that demonstrated that in PDAC the presence of tumor-infiltrating lymphocytes with a high Th2:Th1 cell ratio is associated with worse prognosis [43]. 

Within the TME, 3 distinct stromal compartments in PDAC have been identified that are associated with prognosis—mature, immature and intermediate [15]. These 3 stromal subtypes are determined based on the number of cancer-associated fibroblasts (CAF), the maturity of collage fibers and the density of the stromal matrix. Cases with a “mature” stromal compartment had a dense collagenous stroma and low CAF numbers. Cases with a higher cellular and collagen-poor stroma were labelled “immature”, these cases correlated with worse overall survival, whereas cases that fell in between were categorized as “intermediate” [15]. 

**Figure 1:**
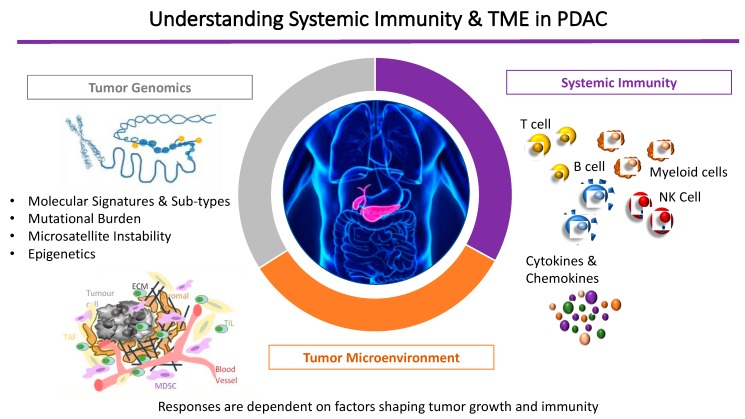
Understanding systemic immunity in the context of the tumor microenvironment and immune-privilege in pancreatic cancer.

In summary, contributing factors to this immune-privileged TME in PDAC include the paucity of T-cell infiltration into the tumor, the low mutational burden in PDAC that produces few immunogenic antigens [12,44] and the physical barrier created by the dense desmoplastic stroma that hinders access of therapeutic monoclonal antibodies, promotes chemokine-mediated exclusion of T cells within the tumor milieu, and reduces T-cell expression of checkpoint receptors [45]. Ultimately, an individual’s responses are dependent on a combination of factors shaping PDAC tumor growth, systemic immunity (innate and adaptive) and the individualized tumor genomic alterations (Figure 1).

To date, only a small subset of PDAC patients with particular characteristics, including a high burden of microsatellite instability (MSI-high) and/or tumors with higher effector T-cell infiltration, have demonstrated a sustained response to programmed cell death-1 (PD-1) blockade with improvement in progression-free and overall survival [29,40]. These responses indicate the potential for effective treatment with immunotherapies in PDAC. Preclinical and early-phase clinical trials are now underway to investigate an array of novel immunotherapy targets as well as rational combinations with existing therapeutic modalities [46,47] (Table 1). Alongside this, the ability to provide a companion immune biomarker may enable these novel immune targets and combination treatments to unlock the full potential of immunotherapy for PDAC patients.

## 4. CTLA-4/CD80 Pathway in Pancreatic Cancer

Checkpoint blockade molecules are present on T cells and serve an inhibitory function. CTLA-4, is homologous to the CD28 receptor. The CTLA-4 prevents differentiation and activation of naïve T cells [48]. The blockade of CTLA-4 by anti-CTLA-4 antibodies results in unrestricted T-cell activation, thus enhancing the T-cell response to the presence of cancer cells [49]. The use of single agent anti-CTLA-4 antibodies in the management of advanced PDAC has shown limited clinical benefit in the majority of pancreatic cancer patients [39,50,51]. Alternative combination therapy approaches are being investigated that can increase T-cell trafficking and infiltration into the tumor microenvironment, thereby sensitizing the PDAC to checkpoint blockade [52].

## 5. PDL1/PD1 Interaction in Pancreatic Cancer

Programmed cell death protein-1 ligand (PDL1) and its receptor, PD1, interact to promote immune tolerance. The blockade of the PD1/PDL1 interaction acts to increase anti-tumor immunity. Results from trials investigating the use of anti-PD1 therapy alone in advanced PDAC have been disappointing [53,54]. However, results from the phase 2 clinical trial led by Le et al., to evaluate PD1 checkpoint blockade with Pembrolizumab in treatment of refractory solid tumors (colorectal and non-colorectal cancers) with MSI-H tumors, demonstrated that in this subset of pancreatic cancer patients, the administration of PD1 therapy resulted in durable responses in patients with 24-month progression-free survival (PFS) rate of 53% and 24-month OS rate of 64% [55]. This study shows that biomarkers for immune response in PDAC exist and are vitally informative. These findings provide a glimmer of hope that other novel immunotherapies, perhaps in combination with different treatment modalities, may be able to overcome resistant mechanisms to immune response in PDAC. To this end, the identification of other immune biomarkers could determine the appropriate immune-based combination therapy and increase the efficacy of both targeted and novel immune therapies at an individualized level to improve therapeutic efficacy and ultimately long-term outcomes. 

## 6. Stimulating the Immune Response in Pancreatic Cancer

Therapeutic vaccine trials involving an array of recombinant constructs, peptides, proteins and whole tumor cells, all designed to prime circulating tumor-specific T cells and attack PDAC, so far have reported negative outcomes [54]. Despite this lack of efficacy, these studies have provided important insight into the immune response in PDAC, confirming that T-cell immunity to tumor-associated self-antigens can be generated and break immune tolerance in PDAC. Furthermore, studies like the phase 2b GVAX pancreas ECLIPSE study demonstrated that it was possible to induce tertiary lymphoid structures and T-cell infiltration in patients [56]. It has been hypothesized that dysfunctional T cells and suboptimal antigen selection resulted in the overall lack of long-term survival in the therapeutic vaccine trials, despite clear evidence of an immunological response in some patients [57,58].

Approaches that combine therapeutic vaccines with checkpoint blockade are being undertaken to investigate whether checkpoint blockade can re-invigorate vaccine-primed T cells to overcome the immunosuppressive inhibition generated by the PDAC tumor microenvironment [59]. 

## 7. Novel Immune Targets of Interest in Pancreatic Cancer

Work by Balli et al. into immune cytolytic activity stratified PDAC patients into molecular subsets. Here they identified groups of PDAC patients that, despite evidence of activated T cells and detectable neoepitope burden, had a complete lack of benefit from anti-PD1 therapies, thus demonstrating that the PDL1/PD1 axis is only one component of the T-cell response mechanism in PDAC [60]. Alternative inhibitory pathways that are highly expressed in PDAC are being investigated in early-phase clinical trials. These include the extracellular enzyme, CD73, which stimulates the release of adenosine, a pro-metastatic and immunosuppressive molecule; inhibitory receptors on T cells analogous to PD1 such as TIM3, TIGIT, and LAG3; and the inhibitory ligand present on myeloid cells, VISTA, which is analogous in function to PD-L1. 

Alternative orthogonal immunotherapeutic approaches of interest in PDAC involve the use of agonistic immunotherapies—which include CD40, OX40 agonists, toll-like receptors (TLR), agonists and stimulator of interferon genes (STING) agonists—and myeloid-based immunotherapies. 

## 8. Agonistic Immunotherapies

### 8.1. CD40 Agonists in Pancreatic Cancer

The co-stimulatory protein, CD40 is expressed by antigen presenting cells including dendritic cells, monocytes, B cells, endothelium, platelets and some tumor cells. The CD40 agonists in combination with gemcitabine have been shown to induce anti-tumor T-cell response and transform macrophages in PDAC from a pro-inflammatory status to a tumoricidal phenotype [61]. Combining CD40 agonists with nab-paclitaxel and gemcitabine further enhanced the T cell-mediated tumor destruction and generated immune memory, not seen with the gemcitabine combination alone [62]. Preclinical models indicate that the combination of CD40 agonist with gemcitabine and nab-paclitaxel can sensitize PDAC to immune checkpoint blockade [63].

### 8.2. OX40 Agonists in Pancreatic Cancer

Co-stimulation by OX40 (CD134) molecule has been shown in preclinical animal models to counteract Treg suppression by activating tumor-infiltrating lymphocytes (TILS) and inducing effector T cell expansion. The OX40 thus provides an alternative strategy by which anti-tumor immunity can be approached. In preclinical murine models, the combination of anti-PD1 therapy with an agonist OX40 antibody promoted T-cell migration into the pancreas tumor, turning a “cold” tumor “hot”. This combined approach significantly enhanced the tumor regression and long-term survival [64]. Clinical trials with MEDI0562, an OX40 agonist, in combination with Tremelimumab (anti-CTLA4 antibody) or Durvalumab (anti-PDL1 antibody) in advanced solid tumors are underway [65] (NCT02705482) (Table 1).

### 8.3. STING Agonists in Pancreatic Cancer

The stimulator of interferon genes (STING) is a transmembrane protein situated in the endoplasmic reticulum. Activation of the cyclic GMP-AMP synthase (cGAS)–STING pathway by direct binding of cyclic dinucleotides (CDNs) triggers a downstream innate immune cascade involving TANK-binding kinase-1/interferon regulatory factor 3 (TBK1/IRF3), nuclear factor-KB (NF-KB) and signal transducer and activator of transcription 6 (STAT6), ultimately resulting in a pro-inflammatory cytokine and type 1 IFN production. This, in turn, primes antigen-presenting cells (APCs) and macrophages to promote an adaptive T-cell response against tumor cells. Preclinical murine models have demonstrated that a combination of an intra-tumorally delivered STING agonist (8803) induces PDAC regression. When combined with either anti-CTLA or anti-PD1 therapy, marked improvement in tumor regression along with a significant improvement in survival was seen compared with single agent STING agonist (8803) therapy alone (*p* = 0.001 vs. *p* = 0.008) [66]. Clinical trials with STING agonist MK-1454 alone or in combination with Pembrolizumab in advanced solid tumors are underway (NCT 03010176) (Table 1).

## 9. Toll-Like Receptor Agonists

Toll-like receptors (TLRs) play a crucial role in discriminating between self and non-self, leading to innate and adaptive immune responses. Toll-like receptors are expressed on both non-immune cells (epithelial cells, fibroblasts and cancer cells) as well as multiple immune cells (B cells, natural killer cells, dendritic cells and macrophages). Toll-like receptors promote the activation of pro-inflammatory cytokines and anti-tumor cytotoxic T lymphocyte production. Many of the TLR family, including TLR2, TLR3, TLR4, TLR7 and TLR8, are expressed in PDAC [16] and influence tumor proliferation, metabolism and dissemination [67,68]. Several different TLR agonists have been evaluated in early-phase clinical trials across multiple advanced solid tumors. Results from these studies have not yet been reported; however, the phase 1 trial of TLR8 Agonist VTX-2337 and cyclophosphamide in patients with metastatic or progressive solid tumors, which included PDAC patients, was terminated early (NCT02650635). The pathophysiology of the various TLRs implicated in PDAC is complex and not limited to the direct effect on cancer cells only. Despite encouraging preclinical data, further research is required to fully comprehend the mechanisms involved in the TLR pathway and how best to translate this into clinical practice. 

## 10. Conclusions

The relationship between inflammation and PDAC is only just beginning to be appreciated. Our knowledge of the components of the tumor microenvironment and their relationship to patient prognosis in PDAC is in its infancy compared to other cancers [8,9]. There are many inherent challenges in applying immunotherapy in PDAC based on the immunosuppressive and immune-privilege nature of the PDAC microenvironment, the tumor heterogeneity and the quality of the immune response. Nevertheless, strategies are being developed to promote T-cell immunity and draw T cells into the PDAC tumor as well as enhance the efficacy of cytotoxic therapies. Combination strategies with different modalities of treatment, and novel immunotherapy targets may hold the key to unlocking the potential of immunotherapy in PDAC. A comprehensive characterization of the relationship between genetic sub-type, immune cell infiltration, and patient-related features could be leveraged for functional studies in animal models as well as used to develop a blood-based stratification biomarker. Identification of protein-based markers linked to treatment and immunotherapy outcome may also enable closer monitoring of a patient response to chemotherapy and immunotherapy, and prediction of clinical outcome. 

## Figures and Tables

**Table 1:** Early phase clinical trials investigating novel immunotherapy targets in pancreatic cancer.

Trial Description	Phase	Target Agent(s)	Target	Active Clinical Trial
**CTLA-4/CD80 Pathway Clinical Trials in Pancreatic Cancer**				
FOLFIRINOX followed by Ipilimumab with allogenic GM-CSF transfected pancreatic tumor vaccine in metastatic pancreatic cancer	2	Ipilimumab	CTLA-4	NCT01896869
Tremelimumab and/or MEDI4736 in combination with stereotactic radiation therapy (SBRT) in unresectable pancreatic cancer	1	Tremelimumab MEDI4736	CTLA-4 PD1	NCT02311361
Tremelimumab and MEDI4736 in combination with hypo-fractionated radiotherapy	1	Tremelimumab MEDI4736	CTLA-4 PD1	NCT02639026
BMS-986205 in combination with Nivolumab and in combination with both Nivolumab and Ipilimumab in advanced cancers	1 and 2	BMS-986205 Nivolumab Ipilimumab	CTLA-4 IDO PD1	NCT02658890
**PD1 Pathway Clinical Trials in Pancreatic Cancer**				
Nivolumab in combination with CAPIRI in advanced pancreatic cancer	1 and 2	Nivolumab	PD1	NCT02423954
Pembrolizumab in combination with gemcitabine and nab-paclitaxel in metastatic pancreatic cancer	1 and 2	Pembrolizumab	PD1	NCT02331251
Neoadjuvant chemoradiation in combination with Pembrolizumab in borderline resectable pancreatic cancer compared to chemoradiation therapy alone	1 and 2	Pembrolizumab	PD1	NCT02305186
Neoadjuvant/Adjuvant GM-CSF secreting allogeneic pancreatic cancer vaccine (CY/GVAX) with or without Nivolumab before and after surgery in resectable pancreatic cancer	1 and 2	GVAX Nivolumab	GVAX PD1	NCT02451982
AMG820 and Pembrolizumab combination in advanced solid tumors	1	AMG820 Pembrolizumab	CSF-1R PD1	NCT02713529
BL8040 as a single agent or in combination with Pembrolizumab in metastatic pancreatic cancer (COMBACT/KEYNOTE-202)	2	BL-8040 Pembrolizumab	CXCR4 PD1	NCT02826486
**Agonistic Immuno-Oncology Pathways in Clinical Trials in Pancreatic Cancer**				
Neoadjuvant RO7009789 alone or in combination with nab-paclitaxel and gemcitabine followed by adjuvant RO7009789 plus nab-paclitaxel and gemcitabine in resectable pancreatic cancer	1	RO7009789	CD40	NCT02588443
MEDI5083 alone or in combination with Durvalumab in advanced solid tumors (with pancreatic cancer cohort)	1	MEDI5083 Durvalumab	CD40 PDL1	NCT03089645
LOAd703 Oncolytic virus therapy for pancreatic cancer	1 and 2	LOAd703	CD40 4-188L	NCT02705196
CCX872-B in combination with FOLFIRINOX in pancreatic cancer patients	1	CCX872-B	CCR2	NCT02345408
MEDI0562, an OX40 agonist, in combination with Tremelimumab (anti-CTLA4 antibody) or Durvalumab (anti-PDL1 antibody) in advanced solid tumors	1	MEDI0562 Tremelimumab Durvalumab	OX40 CTLA-4 PDL1	NCT02705482
MK1454 alone or in combination with Pembrolizumab in advanced solid tumors	1	MK1454 Pembrolizumab	STING	NCT03010176
